# Editorial: Insights in antigen presenting cell biology: 2022

**DOI:** 10.3389/fimmu.2023.1206040

**Published:** 2023-05-10

**Authors:** Peter van Endert

**Affiliations:** ^1^ Université Paris Cité, INSERM, CNRS, Institut Necker Enfants Malades, Paris, France; ^2^ Service Immunologie Biologique, AP-HP, Hôpital Universitaire Necker-Enfants Malades, Paris, France

**Keywords:** antigen processing, antigen presentation, dendritic cell, metabolism, cell culture, migration

This year’s Insights articles comprise an eclectic collection of original articles and reviews concerning dendritic cells (DCs), interference with antigen processing to enhance tumor therapy, and the very broad rules of antigen selection for HLA presentation. Several articles challenge current concepts while others resume or refine established knowledge. In this editorial I briefly present each article, adding some comments designed to stimulate thought and discussion.

D’Amico et al. discuss how modulation of antigen processing might enhance the efficacy of immune checkpoint therapy for cancer that requires recognition of tumor epitopes by CD8^+^ T lymphocytes but so far fails to show efficacy for many patients, especially those with solid tumors. The idea is to increase tumor antigenicity by altering the immunopeptidome displayed by tumor cells. Three points of intervention are considered: the Transporter associated with Antigen Processing (TAP), the chaperone TAP-Binding Protein Related (TAPBPR), and the endoplasmic reticulum aminopeptidases (ERAPs). TAP inhibition has been shown to give rise to immunogenic TEIPP (T cell epitopes associated with impaired peptide processing) activating antitumor T cells, while TAPBPR inhibition can lower the affinity threshold for peptide presentation, and ERAP inhibition result in presentation of epitopes normally destroyed by “over-trimming”, both giving rise to presentation of previously “cryptic” epitopes. However, two major obstacles must be overcome to exploit an altered immunopeptidome: interference must be targeted to tumor cells, and the immunosuppressive tumor microenvironment must be counteracted to allow for CD8^+^ T cell activation and tumor killing.

Karnaukhov et al. revisit the question whether different HLA class I alleles display a preference for presenting peptides derived from proteins with distinct functions. Previous studies examining large peptide sets eluted from HLA proteins had concluded that this is not the case; here the authors argue that the peptide sets might have been too limited to discern such preferences, and consequently base their analysis entirely on algorithm-predicted peptides. Juggling with very large numbers (93 HLA alleles covering 95% of the human population, all 20,000 proteins of the human genome, an average 5 x 10^5^ ligands for each allele), the authors detect proteins yielding significantly more or less ligands than expected for individual HLA alleles. Not surprisingly, these effects are based on the anchor residues selected by the alleles concerned. The preferences detected can be summarized shortly by saying that alleles with hydrophobic anchors prefer membrane proteins, while alleles binding ligands through positively charged residues prefer DNA binding proteins (frequently comprising such residues). The authors also propose the existence of “haplotype compensation”, i.e. the presence in common haplotypes of HLA-A/C or HLA-A/B pairs with opposite preferences (e.g. membrane + DNA binding), ensuring broad coverage of the proteome. Curiously, compensation is not detected for HLA-B/C pairs, although one would expect evolutionary selection of such pairs due to the strong linkage disequilibrium between HLA-B and HLA-C.

The remaining four articles all study DC biology. Wu et al. review current knowledge on metabolic regulation of DC activation and function. Summarized, DC quiescence is maintained by AMPK (AMP-activated protein kinase) itself activated by LKB1 (liver kinase B1) and characterized by glucose metabolization through oxidative phosphorylation. DCs switch to preferential glycolysis upon activation, e.g. triggered by LPS, that is timed in two sequential phases. However, as the authors emphasize, most of the pertinent data were obtained using *in vitro* differentiated murine bone marrow DCs (BM-DCs) or human monocyte-derived DCs, two populations representing native *in vivo* DC populations only partly, thus much work remains to be done on primary conventional DCs (cDCs). Lellahi et al. contribute an original article with practical interest, examining the survival and function of human primary cDCs purified from buffy coats in the presence of cytokines. While >50% of type 1 cDCs (cDC1s) and >90% of cDC2s cultured in any of three standard media undergo apoptosis in 24 hours, addition of individual or combined cytokines (fms-related tyrosine kinase 3 ligand [Flt3-L], granulocyte-macrophage colony-stimulating factor [GM-CSF], interleukin 4 [IL-4]) increases survival strongly; the authors also study activation markers, uptake of FITC-dextran, T cell proliferation and response to Toll-Like Receptor (TLR) ligands. Collectively these results should be of interest to laboratories, for example, preparing DCs for therapeutic purposes in patients.

Specialists will be interested in the original article by Song et al. who develop a machine learning approach to identify migration patterns of BM-DCs confined in agarose gels in the absence of an external stimulus. While studies on innate DC migration have reported switching between diffuse and persistent motility, the machine learning approach used in this study identified three dynamic modes of migration, described as slow-diffusive, slow-persistent, and fast-persistent. These studies will have to be complemented by analysis of DC populations representing primary DCs more closely, e.g. Flt3-L differentiated DCs, and performed in the presence of biological, chemical or physical stimuli. Herbst et al. conclude the series by an opinion-type article challenging current views on antigen acquisition by DCs. Based on results obtained by the group in which Langerhans cells were adoptively transferred into mice expressing YFP in keratinocytes, the authors propose that DCs acquire mRNA from neighboring cells in a contact-dependent manner. To support their hypothesis and a “physiological advantage” of their model, the authors argue that pathogen-invaded cells will be “reluctant” to release antigens, and that other known modes of contact-dependent antigen transfer require “cooperation” by the donor cell (trogocytosis), are limited with respect to antigen size (gap junctions) or hampered by dependence on actin and motor proteins (tunneling nanotubes). According to the authors, the hypothetical mechanism, termed “intracellular monitoring”, could be advantageous in cancer (detection of perturbed metabolic homeostasis and cell stress) and immune tolerance (mRNA from thymic epithelial cells transferred to DCs for negative selection) but also be detrimental (imprinting of tumor infiltrating DCs by a suppressive microenvironment). Clearly, while the hypothesis is interesting, demonstration that the mechanism plays a dominant role in intracellular material acquisition *in vivo* will be required.

## Author contributions

The author confirms being the sole contributor of this work and has approved it for publication.

